# Youth Exposed to Armed Conflict: The Homeroom Teacher as a Protective Agent Promoting Student Resilience

**DOI:** 10.3390/ijerph22081233

**Published:** 2025-08-07

**Authors:** Lia Shur-Kraspin, Michelle Slone, Yaniv Kanat-Maymon

**Affiliations:** 1 School of Psychological Sciences, Tel Aviv University, Tel Aviv 6997801, Israel; liashur4@gmail.com; 2 Baruch Ivcher School of Psychology, Reichman University, Herzliya 4610101, Israel; ykanat@runi.ac.il

**Keywords:** armed conflict, school teacher, teacher self-efficacy, teacher life satisfaction, student resilience, student psychiatric symptoms

## Abstract

Armed conflict poses a significant threat to the mental health of youth worldwide. This study focused on the role of teachers as protective agents fostering resilience among their students. The study examined the moderating effects of teachers’ personal well-being and their efficacy in the school on relations between their students’ armed conflict exposure and student psychiatric symptoms. Participants included 1260 students and their homeroom teachers from 62 8–11th grade classes. Using self-report standardized measures, teachers reported their life satisfaction and sense of efficacy while students reported their armed conflict exposure and psychiatric symptoms. Data were analyzed using a multilevel modeling (MLM) approach. Findings revealed positive correlations between student armed conflict exposure and psychiatric symptoms. In the between-class level of analysis, teacher personal life satisfaction and efficacy in participation in the school system emerged as protective factors for the students, significantly moderating relations between student exposure and their psychiatric symptoms. However, teacher efficacy in class management showed no significant moderating effect on student mental-health difficulties. Results highlight the importance of supporting teachers in conflict-affected environments and emphasize the need for preventive and therapeutic initiatives that prioritize teacher well-being and organizational resources to enhance teachers’ capacity to foster student resilience.

## 1. Introduction

As the number of civilians subjected to armed conflict dramatically rises, the effects of war, armed conflict, and terrorism on the civilian population have become an issue of global concern [1]. Children and adolescents who are compelled to cope with normal developmental tasks while facing violence and instability are at particular high risk of experiencing adverse psychological consequences [2,3,4]. Therefore, it is critical to address the psychological needs of youth in conflict zones. However, the availability of professional resources to do so is often limited.

As part of the effort in tending to the psychological needs of youth, a growing body of research has indicated that schools are an optimal platform to focus on and enhance children’s and adolescents’ resilience [5,6,7,8,9]. More specifically, researchers have shown increasing interest in homeroom teachers and their role in supporting students’ emotional needs, while promoting their ability to adapt and cope with the negative effects of armed conflict [10,11,12,13,14,15]. However, most of the field research tends to overlook teachers’ own emotional state and capacities in these conditions. In areas plagued by violence and instability, teachers are also subject to traumatic exposure, which may undermine their emotional availability to meet the needs of their students [16].

The central aim of the current study was to examine the moderating function of a set of variables that could interact with teachers’ own exposure to traumatic and stressful experiences on their ability to support the resilience of their students. This research goal was translated into a field study that examined the moderating effects of teacher well-being and personal resources on student self-reported measures of psychiatric symptoms in conditions of armed conflict exposure. More specifically, as indicators of teacher well-being and personal resources, the study focused on perceptions of teachers of their own life satisfaction and self-efficacy.

### 1.1. Risk and Resilience in the Face of Protracted Armed Conflict

Over the course of the 20th century, war and armed conflict have changed dramatically, generating a major impact on individuals of all ages [17]. Contemporary forms of war and armed conflict occur in the midst of civil society, specifically target civilians [18], and force many populations around the globe to live under ongoing threat and danger [19,20]. These conditions expose individuals to a wide range of extremely stressful and traumatic experiences, including the threat or witnessing of death or serious injuries, property damage, hearing explosions or gunfire, and forced relocation and separation from family and friends [15]. Moreover, events of armed conflict not only inflict physical and mental repercussions on those directly exposed, but they also deeply undermine all sense of security and predictability among the general public [21]. Therefore, growing up in an environment stricken by war, violent armed conflict, and terrorism can exert a heavy psychological toll on the mental health and development of children and adolescents [4].

The literature shows substantial support for the links between both direct and indirect exposure to armed conflict events and a range of both major syndromes and subthreshold symptoms of post-traumatic stress disorder [22,23,24,25], various anxiety disorders [26,27,28,29], depression [2,26,30,31], somatic complaints [32,33,34], general distress symptoms [21,22,35] and more. However, alongside the extensive body of research suggesting that exposure to armed conflict can lead to a variety of developmental impairments and psychiatric disorders [36,37,38], previous research has shown wide heterogeneity in mental health outcomes following exposure. Findings can be conceptualized as a spectrum, ranging from severe impairment to minimal effect [39]. These diverse findings have led to the conclusion that exposure to a traumatic event alone is insufficient to predict its trajectory [40] and have illuminated the role played by both characteristics of the trauma, together with various risk and protective factors that moderate the effects of trauma exposure [41].

Resilience has been defined as an individual’s capacity to maintain relatively stable psychological and physical functioning, despite exposure to a potentially highly disruptive event [42]. Identifying predictors of resilience is essential for creating interventions and for investing resources aimed at improving the mental health of children and adolescents [43,44]. While the initial research on resilience focused mainly on exploring individual traits and characteristics that promote resilience [45], the exploration of protective factors has broadened beyond the individual level, taking into account the influence of various social-ecological environments on children’s and adolescents’ adaptation [46].

Accordingly, Betancourt and Khan [47] view traumatic exposure outcomes as a result of dynamic processes of interactions between risk and protective factors operating at different levels of the child’s social environment. Not only can the direct exposure of children and adolescents to armed conflict events endanger their physical and mental health, but these adversities can also have a negative impact on their extended support systems, such as family, social, and economic conditions, and therefore can affect the quality of care and protection provided [48,49]. On the other hand, positive adjustment and protective capacities of prominent figures and systems in the social environment of youth, such as a supportive family and access to school, can mitigate the negative effects of war exposure [4,9,35,50,51]. Therefore, in order to achieve a more comprehensive understanding of the effects of armed conflict, there is a need to examine both protective capacities and deficits of key individuals and systems in the environment of youth [1].

### 1.2. The School and Homeroom Teacher in Times of Crisis

The school is situated at the heart of the community and constitutes an integral part of the lives of youth. In a chaotic or threatening environment, schools may provide a secure base in which children and adolescents can benefit from circles of support [52]. More specifically, homeroom teachers are well-positioned to serve the vital role of recognizing difficulties, tending to emotional needs, and serving as mediators of clinical principles to their students [53].

The relational dynamic between teachers and students has become a central focus in educational research, consistently associated with children’s school adjustment and developmental success [54,55]. Attachment theory [56] provides a relevant framework for interpreting these findings, emphasizing that emotionally available attachment figures foster a sense of security and self-worth, reinforcing the expectation that support will be accessible in times of need. Early caregiving relationships are foundational in shaping children’s internal working models of self and others, which, in turn, influence their ability to form secure and nurturing relationships later in life. Although teacher–student relationships are typically less emotionally intense and enduring than those with primary caregivers, research suggests they can still encompass attachment-related features, positioning teachers as contemporary attachment figures [57,58,59]. When teachers are emotionally attuned and available, they may function as a secure base, helping students regulate emotions, explore their environment, and adapt to challenges [60]. Empirical studies further support the importance of such bonds, showing that students who perceive their teachers as emotionally responsive report greater positive affect [61], stronger coherence, and lower levels of loneliness [59,62], while teacher rejection has been linked to elevated externalizing and internalizing behavioral problems [61,63].

In the field of trauma exposure care, researchers have focused on two main aspects of teacher support. First, several studies have revealed that positive teacher-student relationships and teacher support can function as protective factors for children and adolescents facing various adversities [9,64,65,66]. Second, many studies have indicated the efficacy of various teacher-delivered interventions to mitigate negative effects of exposure to traumatic events [11,13,14,15,67,68].

While research in the field of trauma exposure care illuminates the possible beneficial influence that teachers can exert on student adaptation [9,10,11,13,14,15,67,68], teachers’ own emotional states and resources have scarcely been taken into account. Teachers have a strong impact on their students’ well-being and personal resources, not solely through the content that teachers impart or the classroom activities, but through their management of the classroom; the ways in which they guide social and emotional functioning, and their connection with their students. All of these daily interactions may be deeply affected by teachers’ own well-being, alongside available personal resources and capacities [69].

Recent studies in the field of education provide initial support that teachers’ own well-being and personal resources can lead to beneficial effects on students’ social-emotional outcomes, whereas distressed teachers can compromise them. Findings indicate positive associations between various indicators of teacher well-being and social-emotional resources and student adjustment, together with findings that demonstrate positive associations between indicators of teacher distress and student maladjustment [70,71,72,73,74,75]. In conditions of armed conflict, exploring possible effects of teachers’ well-being and emotional resources on students’ adaptation is of great importance, as teachers themselves may experience high levels of distress and a depletion of resources that may affect their ability to be emotionally available to meet the needs of their students [16]. However, little is known about the effects of teachers’ mental states and resources on their students during times of armed conflict. Therefore, the current study focused on teacher well-being and personal resources as possible moderators of the consequences of student exposure to armed conflict. More specifically, as indicators of teacher well-being and personal resources, the current study focused on perceptions of teachers of their own life satisfaction and self-efficacy.

#### 1.2.1. Teacher Life Satisfaction

Life satisfaction is viewed as a central indicator of well-being [76] and is defined as the cognitive evaluation of overall satisfaction with life as a whole [77]. The positive subjective experience of life satisfaction has been shown to relate to various positive life outcomes, among which are: better physical health and longevity [78,79,80], work-related functioning [76,81,82,83], and social relationships and behaviors [84,85]. Moreover, longitudinal studies have suggested that life satisfaction is not merely a result of positive life experiences, but also a significant antecedent of positive life outcomes [86,87,88], and can operate as a buffer, mitigating the effects of stressful life experiences [89,90].

The possibility that one’s own life satisfaction may also have beneficial effects on others has not been extensively and empirically studied [75]. However, in the educational field, several preliminary studies point to the potential positive effects that teachers’ life satisfaction can exert on their functioning and thereby on their students. In a longitudinal study of teachers in the “Teach for America Program”, teachers’ life satisfaction predicted the academic gains of their students during the course of the year. The authors suggested that teachers with higher life satisfaction may be more skillful in arousing and engaging their students due to their energetic style and positive attitudes [91]. Another study has shown that teachers with higher life satisfaction manage to create a supportive classroom environment that encourages students to help and share with one another [75].

#### 1.2.2. Teacher Sense of Self-Efficacy

Self-efficacy is defined as individuals’ beliefs regarding their abilities to reach desired outcomes in specific contexts [92]. In the educational field, the concept of teacher self-efficacy has been extensively studied and has been shown to be a significant determinant of a broad range of adaptation and performance outcomes related to the classroom environment [93].

Links between teacher self-efficacy and factors underlying teacher psychological well-being have been extensively and consistently demonstrated [93,94,95,96,97,98,99]. Teachers with lower levels of efficacy beliefs are presumed to experience more anxiety regarding their ability to deal with their job requirements and may not feel sufficiently confident to cope with challenges, all of which may lead to higher levels of stress, depression, and emotional exhaustion [97].

While less extensively studied, teachers’ self-efficacy beliefs are also assumed to affect their educational and instructional quality. According to Bandura [100], individuals’ behaviors are rooted in both the expectancy that a certain behavior will lead to a certain outcome and the belief that one has the ability to produce such behavior. Therefore, Bandura [100] suggested that self-efficacy beliefs affect the initiation of behaviors, the amount of effort exerted in pursuing those behaviors, and their persistence when facing difficulties [100]. Correspondingly, findings have indicated that highly efficacious teachers tend to be more proactive and effective in coping with students’ behavioral problems and student-teacher conflicts [101,102,103,104], establish a more caring and close relationships with students [105], and create more positive classroom climates characterized by support, effective use of time and enthusiasm [106].

These findings suggest that self-efficacious teachers are likely to be more emotionally available and create a more beneficial and supportive learning environment, which can affect student adjustment. Empirical evidence for this view arises from a large-scale study that found that students with teachers who have high job-related efficacy reported better quality of school life [107]. However, studies that examined teacher self-efficacy as a predictor of student psychosocial outcomes are scarce. In the context of an ongoing armed conflict that involves constant instability and enforces teachers to cope with new challenges and requirements, self-efficacy beliefs may play a vital role in teachers’ ability to cope and provide support for their students.

### 1.3. The Present Study

Although research in the field of trauma exposure care has highlighted the beneficial effects that homeroom teachers can exert on students during war and armed conflict periods, the characteristics of teachers that may influence their ability to be a protective agent promoting resilience have yet to be explored. In view of these considerations, the present study aimed to examine the moderating role of teachers’ well-being and personal resources on the relation between students’ armed conflict exposure and psychiatric symptoms. Specifically, the study focused on teachers’ perceptions of their own life satisfaction and self-efficacy.

The goal of the present study was translated into a cross-sectional field study that included self-reported measures from both students and their homeroom teachers. Students’ war exposure and psychiatric symptoms were measured using self-report questionnaires answered by each student. Teacher life satisfaction and self-efficacy were reported by the homeroom teachers.

The research is grounded in the context of education in Israel, a population heavily affected by the Middle East conflicts. Education in Israel is compulsory from age 3 to the completion of high school at age 18, and most students attend public schools, which are tuition-free. The national education system is divided into several distinct sectors. This study focuses on the state-secular sector, in which the majority of students come from the secular Jewish population. Within this sector, students typically enter middle school in Grade 7 and transition to upper secondary school in Grade 10. Compared to elementary schools, secondary schools in Israel are relatively large and characterized by greater socioeconomic diversity, as well as curricular differentiation, including grouping by ability level in core subjects (e.g., mathematics and English), and individually selected tracks from a range of options [108]. Despite this differentiation, most secondary schools assign each student to a homeroom class, led by a designated homeroom teacher, where students typically study several general subjects together [109]. While there is no formal definition specifying the total number of hours homeroom teachers are required to teach in their class each week, the homeroom teacher in Israel plays a central role across all levels of the education system, serving as a key figure responsible for students’ holistic well-being and overall functioning [110]. In addition to instructional and pedagogical duties, the homeroom teacher is tasked with a broad set of responsibilities aimed at monitoring and facilitating students’ behavioral, emotional, moral, and social well-being [111,112]. As such, this provides a suitable framework for the current study.

**Figure 1:**
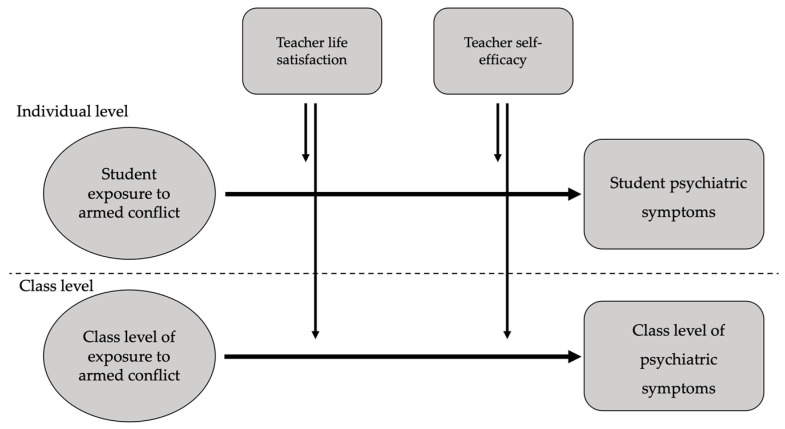
Theoretical model of the study.

Given the nested data structure in the current study and its focus on examining the effects of teacher characteristics on student resilience, a multilevel framework was applied. Multilevel analysis accounts for the interdependency of students in the same classroom, the way students may affect one another, and the way students may be influenced by shared environmental variables, such as the characteristics of their teacher [113]. When examining ways in which teacher characteristics contribute to the prediction of student outcomes, an important consideration lies in specifying the hierarchical level of analysis (i.e., student level or class level) at which relations are expected to hold [114], since relations at one level of analysis may not hold at another level [115]. For example, teacher well-being and personal resources may have a differential effect on student responses to traumatic exposure. Some students may be more reactive than others to supportive interactions with teachers or beneficial modelling, perhaps among those with low support sources outside the school system. Therefore, students may experience their teachers’ well-being and personal resources differently, creating considerable within-class variation. In this case, if teacher well-being and personal resources do have a moderating effect on the relations between student exposure to armed conflict and psychiatric symptoms, it will more likely be evident at the within-class level (i.e., student level) of analysis. Alternatively, teacher well-being and personal resources may exert influence on the class as a whole, for example, by creating a safe class climate that is experienced relatively uniformly across students within that classroom, having a similar mitigating effect on their response to the traumatic exposure. In the latter scenario, the moderating effect of teacher well-being and personal resources on the relation between student exposure to armed conflict and psychiatric symptoms will be more cohesive among students within a classroom, but may create differences between classes that would be evident at the between-class level of analysis (i.e., class level). Both effects can also exist simultaneously. Therefore, in the current study, teacher well-being and personal resources indicators of teacher life satisfaction and self-efficacy were reported by teachers, but their moderating effects were examined on the relation between armed conflict exposure and psychiatric symptoms both at the within-class level and at the between-class level. The goal of the present study and its multilevel model are presented in Figure 1, which outlines the theoretical model of the study.

### 1.4. Hypotheses

In line with the presented theoretical rationale, the study advanced two hypotheses. The first hypothesis predicted a direct relationship between student exposure to armed conflict and psychiatric symptoms, with students exposed to greater severity of armed conflict events expected to report higher levels of psychological distress and post-traumatic symptoms.

The second hypothesis predicted that teacher life satisfaction and self-efficacy would moderate the relationship between students’ armed conflict exposure and their psychological distress and post-traumatic symptoms.

## 2. Materials and Methods

### 2.1. Participants

Participants were 1260 students and their homeroom teachers from 62 8th to 11th grade classes at seven schools across diverse geographic areas in Israel. The student sample included 602 boys and 648 girls (in 10 cases, gender was not reported), aged 12 to 18 (M = 15.48; SD = 1.253). The majority of students were Jewish (95.4%), with 4.2% reporting Orthodox adherence, 36.2% traditional, 56.1% secular, and 3.5% marked “Other”. The homeroom teacher sample included 62 teachers, aged 29–66 (M = 42.13; SD = 8.119), with 43 females and 19 males. Teachers’ sample gender distribution roughly represents the gender distribution in the teaching profession workforce in Israel, which is composed of a majority of 81.3% female and a minority of 18.7% male [116]. A total of 63.9% of the teachers held a Master’s degree or higher and 36.1% held a Bachelor’s degree. Further, 14.5% of the teachers had 2–5 years of experience in the teaching profession, 33.9% had 6–10 years of experience, and 51.6% had above 10 years of experience.

### 2.2. Instruments

#### 2.2.1. Student Socio-Demographic Background

Socio-demographic details were collected using a custom socio-demographic questionnaire developed for the purpose of the present study. The questionnaire contained questions regarding gender, student grade, country of birth, religion, religiosity, and marital status of parents.

#### 2.2.2. Student Exposure to Armed Conflict Events

To assess the severity of exposure to armed conflict violence and terrorist events, participants completed a modified version of the Political Life Event Scale (PLE). The PLE includes 20 event items [117], and participants are asked to indicate whether they have been exposed to each event and to rate the subjective impact of each exposed event on a 5-point Likert scale (1 = not at all, 5 = very much). The items include mild events (e.g., being present in a situation where there is a suspected dangerous explosive), moderate events (e.g., the injury of a friend or acquaintance as a result of military or armed conflict), and severe events (e.g., the death of a relative as a result of an armed conflict event). The total score for the severity of armed conflict exposure is calculated by summing the impact scores of all items marked as positive for exposure.

In the current study, a modified version of the PLE was used, which included only moderate and severe event items. This adjustment was made because, over time, mild items have become universally experienced in the region (e.g., “security check on entering a public place”). Additionally, four new items were added to reflect armed conflict events occurring in recent years (e.g., “I was exposed to a kite/balloon carrying explosives”).

The PLE scale has been widely used and studies have shown high predictive validity for communities in conflict areas [29], good discriminant validity with cross-nationality transferability for Jewish and Arab Israeli youth [118], as well as for Palestinian youth [119]. Test–retest scores range from *r* = 0.86 to *r* = 0.94 [32]. In the current study, the PLE score demonstrated good internal consistency (α = 0.83).

#### 2.2.3. Student Psychological Distress

Students’ psychological distress was assessed using the Brief Symptoms Inventory (BSI-18). The BSI-18 scale is an effective screening tool for psychological distress and psychiatric disorders [120]. In this inventory, 18 symptoms are rated on a 5-point Likert scale (0—not at all, 4—very much). The questionnaire yields three subscales: anxiety, depression, and somatization, and a general distress index (GSI), calculated as the average of ratings assigned to all symptoms and considered to be the best indicator of depth of distress [120]. The scale has shown internal consistencies ranging from 0.74 to 0.89 [120]. The BSI-18 is an abbreviated version of the BSI (53 items), which has demonstrated reliability and validity in numerous research studies, including samples of non-clinical adolescents [121]. The BSI can be appropriately used from the age of 12 and has been widely used in Israel with the current study age group [32,122]. In the present study, internal consistencies were high with α = 0.89 for the anxiety subscale, α = 0.86 for the depression subscale, α = 0.86 for the somatization subscale, and α = 0.94 for the general distress index.

#### 2.2.4. Student Post-Traumatic Symptoms

Post-traumatic stress symptoms were measured by the CPSS-SR, a self-report measure for children aged 8–18 [123]. In the present study, the CPSS-5 was used, a modified version that maps onto the diagnostic criteria for PTSD in DSM-V [124]. The scale includes 27 items, requesting respondents to rate the frequency of experiencing each of the 20 DSM-V PTSD symptoms on a 5-point Likert scale, ranging from 0 (not at all) to 4 (6 times a week or more). In addition, seven areas of possible functional impairment are rated on the same 5-point Likert scale. The questionnaire yields a severity score that is calculated by summing the ratings of the 20 items assessing DSM-V PTSD symptoms that range from 0 to 80. The seven items assessing impairment of endorsed symptoms on daily functioning are not included in the overall severity score. The CPSS-5 has demonstrated excellent reliability and validity in a diverse sample of youth (Cronbach’s alpha = 0.92; test–retest reliability r = 0.80) [125]. The original questionnaire assesses post-traumatic stress symptoms caused by exposure to a specific stressful event. For the current study, the questionnaire was adapted to suit individuals exposed both directly and indirectly to events related to the armed conflict in Israel. As part of this adaptation, one item was omitted. In the current study, the questionnaire produced high internal consistency (α = 0.91).

#### 2.2.5. Teacher Socio-Demographic Background

Teacher socio-demographic details were collected using a custom socio-demographic questionnaire developed for the purpose of the present study. The questionnaire includes questions regarding gender, age, marital status, years of experience in the teaching profession, and level of academic degree.

#### 2.2.6. Teacher Satisfaction with Life Scale

In order to examine teachers’ life satisfaction, the five-item self-report Satisfaction with Life Scale (SWLS) questionnaire was used [126]. Respondents are asked to mark their agreement with each item on a 7-point Likert scale (1—not at all, 7—very much). This scale has been widely used with a variety of populations [77] and is reported to have good internal consistency (α = 0.87) and good test–retest reliability (*r* = 0.82) [126]. Internal consistency in the present study was good (α = 0.86).

#### 2.2.7. Teacher Self-Efficacy

Teacher efficacy was evaluated using the Teacher Self-Efficacy Scale [127] which yields two subscales: (a) classroom context efficacy including sense of professional efficacy pertaining to teaching, educating and motivating students as well as conducting interactions with students and (b) school context efficacy including involvement in school activities, participation in decision-making, influencing school organizational politics and ability to attain support from the school as a system. Each item describes an ability in one of the two function areas. Respondents are asked to rate the frequency with which they perceive themselves as possessing the described ability in the past month on a 6-point Likert scale (1—never, 6—always) [127]. The scale is reported to have satisfactory internal consistency for each subscale separately (α = 0.85 and 0.85). In the present study, internal consistencies for each subscale were also good (α = 0.89 for the classroom context efficacy subscale, and α = 0.86 for the school context efficacy subscale).

### 2.3. Procedure

After obtaining authorization from the Israeli Ministry of Education and the Tel Aviv University Ethics Committee, school principals were approached to take part in the study. While 11 school principals expressed willingness to participate, one school was disqualified due to its sociodemographic characteristics, and in three schools, the study could not be conducted due to changes to the annual school schedule. Therefore, the final sample included 7 schools. After receiving approval from school principals, teachers, and parents of students under the age of 16 provided written informed consent, while parents of students above the age of 16 were informed that they could object to their child’s participation in the study. No incentives were provided to participants for their participation. In total, 9 teachers and 39 students declined to participate. Administration of questionnaires was conducted by trained BA Psychology students. Students and their homeroom teachers completed the questionnaire batteries at the same time in their 50-minute home classes. Since the research required matching data from students and their homeroom teacher, each student and teacher were assigned a class code (a code shared by all students in the same class and their homeroom teacher) to write on the questionnaires. In order to ensure the confidentiality of the participating students, no additional identifying information was collected in the research questionnaires and students were explicitly instructed not to include any identifying details. Furthermore, access to the completed questionnaires was restricted exclusively to the research team and school personnel had no access to the data. Participants were requested to answer questionnaires manually or via the Qualtrics software 2019–2020 using their personal mobile phones, according to their personal preference. Missing data ranged from 0.2% to 4.5% in the students’ main variables and 1.6% to 3.2% in the teachers’ main variables. During the administration time, a research assistant was present and available for assistance. Following completion of questionnaires, each student received a letter which included information regarding possible support providers in the case of distress as a result of answering the research questions. In addition, the research was conducted in collaboration with the school counselors. Soon after the administration was completed, the research team reviewed the questionnaires and school counselors were provided with information regarding high levels of distress identified in the data. This information was conveyed in an anonymous manner such that counselors were informed only of the number of students per class who were identified as potentially at high risk, without disclosing any personal identifiers. Despite its anonymous nature of this information, sharing it with the counselors enabled sensitive intervention in the classroom. Awareness of the presence of students experiencing significant emotional distress allowed counselors to increase their availability, observe classroom dynamics more closely, and create opportunities for at-risk students to seek support, thereby promoting student well-being while upholding their privacy.

### 2.4. Analytical Strategy

The current study was conducted in a school setting where the 1260 participating students (level 1) are nested within 62 classrooms (level 2). Intra-class coefficient (ICC) values for the dependent measures ranged between 0.01 and 0.03, which requires a multilevel modeling (MLM) approach to test the research hypotheses [128]. Moreover, the MLM approach offers the advantage of testing cross-level interactions, as hypothesized in the current study. Specifically, it allows for examining how the association between students’ exposure to armed conflict and their psychiatric symptomatology (a Level 1 relationship) varies as a function of teacher characteristics (a Level 2 variable). An MLM approach was applied using the SPSS mixed procedure (SPSS Ver.27).

To test the research hypotheses, five series of multilevel models were constructed, one per dependent variable (i.e., student general distress, anxiety, depression, somatization, and post-traumatic stress symptoms). The variance in the predictor, exposure to armed conflict (PLE score), was decomposed into within- and between-class components. Within-class PLE was group-centered and class means (aggregated scores) for PLE were grand-centered and introduced at the class level [129]. Given the complexity of the study and limited sample size, in each analysis, only a single moderating variable was introduced. To ease the interpretation of the MLM coefficients, all analyses were conducted in three phases. The first phase tested the main effect of student PLE. In the second phase, the main effect of the moderating variables (i.e., teacher life satisfaction, teacher classroom context efficacy, and teacher school context efficacy) was added to the model. In the third step, the interaction between student PLE and the relevant moderating variable was added.

All models included the moderation effects at both the within- and between-class levels. That is, how the association between student PLE and student psychiatric symptoms measures was moderated and how the association between average class PLE and average class psychiatric symptoms measures was moderated. To probe the significant interactions, follow-up simple slopes analyses were conducted as customary when analyzing interactions in regression-like models [130]. Specifically, the effect of PLE on the outcome of interest was examined at three levels of the moderator variable (i.e., Mean − 1SD, Mean, Mean + 1SD). Missing data were handled using the maximum likelihood method, a state-of-the-art method for obtaining estimates of the parameters [131]. The method uses all available data to calculate maximum likelihood parameter estimates with standard errors that are robust to non-normality.

Lastly, in order to account for possible covariations between socio-demographic factors on the main research variables, the socio-demographic data were introduced as covariations. Based on previous studies, four factors that have been found consistently relevant to the study of armed conflict consequences and student-teacher relationship were examined as possible confounders: student gender [23,132], grade level [10,133], teacher gender [134], and teacher seniority [135,136]. All of the analyses of the study were performed with and without controlling for socio-demographic variables. This is according to the claim [137] that comparing with and without covariate analyses enables non-biased transparency in the extent to which findings are reliant on the presence of a covariate.

## 3. Results

### 3.1. Descriptive Statistics and Bivariate Correlations

Means, standard deviations, and bivariate correlations of the study variables and socio-demographic variables are presented in Table 1. As can be seen, significant correlations emerged between the socio-demographic variables and most measures of student psychiatric symptoms. Therefore, the effects of the socio-demographic variables on the other variables in the study were further examined, as described in the robustness analysis subsection.

### 3.2. Hypotheses Testing

#### 3.2.1. Testing Hypothesis 1: Student PLE and Psychiatric Symptoms

As the first hypothesis predicted, at the within-class level, student PLE was significantly associated with each measure of student psychiatric symptoms (see the upper part of Table 2). These findings suggest that students who reported higher levels of PLE also reported higher levels of general distress, anxiety, depression, somatization, and post-traumatic stress symptoms. Significant associations were also found at the between-class level (bottom part of Table 2), classes with higher average class PLE had higher average class general distress, anxiety, and post-traumatic stress. However, average class PLE was not associated with average class depression and somatization.

#### 3.2.2. Testing Hypothesis 2: Homeroom Teacher Life Satisfaction and Self-Efficacy as Protective Factors

The second hypothesis predicted that high teacher life satisfaction, classroom context efficacy, and school context efficacy will serve as protective factors and thus weaken the correlations between student PLE and student psychiatric symptoms measures. The moderating effects of teacher life satisfaction, classroom context efficacy, and school context efficacy were examined both at the within- and between-class levels, in a series of multilevel modelling (MLM) analyses.

(1) Teacher life satisfaction as a protective factor

Measures of student general distress, depression, and post-traumatic stress symptoms were predicted by teacher life satisfaction (B = −0.011, SE = 0.005, *p* < 0.05; B = −0.016, SE = 0.005, *p* < 0.05; and B = −0.161, SE = 0.072, *p* < 0.05, respectively). These findings indicate that classes in which homeroom teachers reported higher levels of life satisfaction had lower average class general distress, depression, and post-traumatic stress symptoms. However, no significant main effects emerged for teacher life satisfaction on student anxiety and somatization. In the next step, the teacher life satisfaction moderating effect was examined. At the within-class level, no significant interaction effects emerged. However, at the between-class level, results yielded three significant interactions between student PLE and teacher life satisfaction on student general distress (B = −0.001, SE = 0.0007, *p* < 0.05), depression (B = −0.001, SE = 0.0007, *p* < 0.05) and somatization levels (B = −0.001, SE = 0.0007, *p* < 0.05).

**Figure 2:**
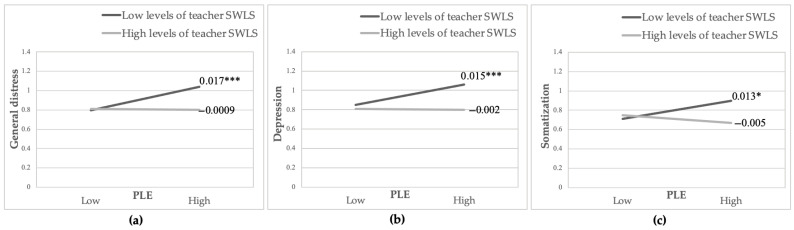
* *p* < 0.05, *** *p* < 0.001. Note: PLE—Political Life Event; SWLS—Satisfaction with Life Scale. (**a**) The interaction effect that emerged between student PLE and teacher life satisfaction on student general distress, between-class level analysis. (**b**) The interaction effect that emerged between student PLE and teacher life satisfaction on student depression, between-class level analysis. (**c**) The interaction effect that emerged between student PLE and teacher life satisfaction on student somatization, between-class level analysis.

In order to probe the significant interactions, follow-up simple slopes analyses were conducted. Average class general distress, depression, and somatization were examined as a function of average class PLE, at 1 SD above and below the mean of the teacher life satisfaction level [138]. All results confirmed the hypothesized pattern of interactions. As seen in Figure 2a, for classes in which homeroom teachers reported low life satisfaction, higher average class PLE was associated with higher average class general distress (B = 0.017, SE = 0.003, *p* < 0.001). However, this association was not significant for classes in which homeroom teachers reported high life satisfaction (B = −0.0009, SE = 0.002, *p* = 0.646). Thus, high teacher life satisfaction weakens the effects of PLE on the general distress levels at the between-class level. A similar pattern of effects emerged for student depression and somatization symptoms. As can be seen in Figure 2b, for classes in which homeroom teachers reported low life satisfaction, a higher average class PLE was associated with a higher average class depression level (B = 0.015, SE = 0.003, *p* < 0.001). Yet, this association was not significant for classes in which homeroom teachers reported high life satisfaction (B = −0.002, SE = 0.002, *p* = 0.479). Similarly, for classes in which homeroom teachers reported low life satisfaction, a significant positive correlation emerged between average class PLE and average class somatization level (B = 0.013, SE = 0.006, *p* = 0.046), but this correlation was not significant for classes in which homeroom teachers reported high life satisfaction (B = −0.005, SE = 0.007, *p* = 0.463; see Figure 2c).

(2) Teacher efficacy as a protective factor

Teacher classroom context efficacy was negatively associated with student general distress (B = −0.130, SE = 0.057, *p* < 0.05), depression (B = −0.148, SE = 0.063, *p* < 0.05), and somatization levels (B = −0.125, SE = 0.058, *p* < 0.05). These findings indicate that classes in which homeroom teachers reported higher levels of classroom context efficacy had lower average class general distress, depression, and somatization. However, no significant main effects emerged for teacher classroom context efficacy on student anxiety and student post-traumatic stress symptoms. In addition, contrary to prediction, no significant interactions emerged between student PLE and teacher classroom context efficacy on all student psychiatric symptoms measures, both at the within- and between-class levels of analyses.

With regards to teacher school context efficacy, while no main effects emerged for teacher school context efficacy on student psychiatric symptoms measures, two significant interactions were found between student PLE and teacher school context efficacy on student general distress level (B = −0.014, SE = 0.006, *p* < 0.05), and student depression level (B = −0.017, SE = 0.006, *p* < 0.05) at the between-class level.

**Figure 3:**
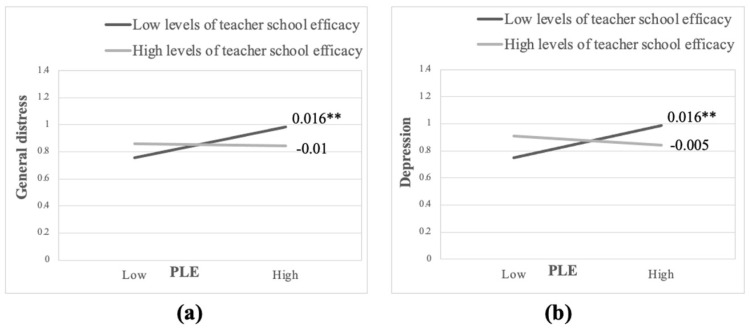
** *p* < 0.01. Note: PLE—Political Life Events; SWLS—Satisfaction with Life Scale. (**a**) The interaction effect that emerged between student PLE and teacher school context efficacy on student general distress, between-class level analysis. (**b**) The interaction effect that emerged between student PLE and teacher school context efficacy on student depression, between-class level analysis.

As can be seen in Figure 3a, in line with the second hypothesis, for classes in which homeroom teachers reported low school context efficacy higher average class PLE was related to a higher average class general distress (B = 0.016, SE = 0.005, *p* = 0.001). Conversely, classes in which homeroom teachers reported high levels of school context efficacy did not show an increase in average class general distress with higher average class PLE (B = −0.001, SE = 0.005, *p* = 0.853). The same pattern of results was found for student depression (see Figure 3b). For classes in which homeroom teachers reported low school context efficacy, higher average class PLE was associated with higher average class depression (B = 0.016, SE = 0.005, *p* < 0.001), whereas this association was not significant for classes in which homeroom teachers reported high levels of school context efficacy (B = −0.005, SE = 0.005, *p* = 0.333).

### 3.3. Robustness Analysis

In order to examine the robustness of the findings, possible confounding effects of the socio-demographic variables were examined. In the first stage, a series of preliminary analyses was conducted, designed to examine which of the socio-demographic variables (i.e., student gender, grade level, teacher gender, and teacher seniority) had a significant effect on the outcome variables over and above the effects of student PLE. Only student gender had a consistently significant effect on all of the dependent variables of the study. Therefore, we examined whether the study’s key findings remained significant when controlling for student gender. All of the study’s main effects and interactions remained significant, apart from the interaction between student PLE and teacher school context efficacy on average class general distress that became only marginally significant with the inclusion of student gender as a covariate (B = −0.009, SE = 0.005, *p* = 0.086).

## 4. Discussion

The present study focused on the role of homeroom teachers in areas plagued by armed conflict, and aimed to explore teacher characteristics that may influence their ability to be a protective agent in promoting resilience for their students. The study examined the moderating roles of teachers’ well-being and personal resources, which were operationalized as perceptions of teachers of their own life satisfaction and self-efficacy on students’ psychiatric symptoms in conditions of armed conflict.

### 4.1. Exposure to Armed Conflict and Youth Psychiatric Symptoms

The first hypothesis of the study predicted that greater severity of student armed conflict exposure would be directly related to student psychiatric symptoms indices. At the within-class level of analysis (i.e., student level), this hypothesis was confirmed. These findings are congruent with previous studies, which indicate increased psychiatric symptoms following high levels of exposure to terrorism and armed conflict [25,29,36,139,140,141,142,143], and attest to the harmful effects of living under protracted conflict and war. As opposed to the traditional focus on post-traumatic stress symptoms as an outcome of armed conflict exposure, the results of the current study highlight that these threatening and dangerous experiences may lead to a wide variety of symptomology.

In addition, the multilevel method of analysis and decomposing the variance of the severity of armed conflict exposure score (i.e., PLE score) into its within- and between-class components yielded another interesting finding. Results revealed not only associations between severity of exposure to armed conflict and psychiatric symptoms at the within-class level of analysis, but also at the between-class level. The class average of armed conflict exposure correlated with the class average of general distress, anxiety, and post-traumatic stress symptoms, when controlling for the within-class variance of exposure to armed conflict. In other words, these findings suggest that regardless of personal exposure to armed conflict, being part of a group characterized by great severity of exposure to conflict-related events placed students at greater risk for suffering from general distress, anxiety, and post-traumatic stress symptoms. Hence, these results may imply that in order to understand children and adolescents’ reactions to armed conflict events, it is insufficient to examine only their own personal exposure. Rather, there is a need to consider the specific context, namely, their immediate environment. While the mechanisms underlying these results are beyond the scope of the current study, a possible explanation could be that living in an environment in which exposure to armed conflict events is commonplace creates a feeling of continuous instability and fear that interferes with mental health, even if the individual’s exposure is mild. In this manner, the findings correspond with the literature, which highlights the possible deleterious effects of indirect exposure to armed conflict.

### 4.2. The Moderating Role of Homeroom Teacher Life-Satisfaction and Self-Efficacy

The second hypothesis of the study predicted that homeroom teachers’ perceptions of their own life satisfaction, class context efficacy, and school context efficacy would moderate the association between severity of student armed conflict exposure and student psychiatric symptoms. This hypothesis was partially confirmed, as explained below, with teacher life satisfaction and teacher school context efficacy serving as protective factors that enabled teachers to support their students’ resilience.

In classes in which homeroom teachers reported low levels of life satisfaction, significant associations were found between class average of armed conflict exposure and class average of general distress, depression, and somatization. However, these associations were not significant in classes in which homeroom teachers reported high life satisfaction. These findings suggest that at the between-class level of analysis, homeroom teacher life satisfaction acts as a protective factor in mitigating students’ general distress, depression, and somatization.

These results support the literature on life satisfaction, indicating that the positive subjective experience of life satisfaction is significantly associated with positive life outcomes [86,87,88,89]. More specifically, these findings highlight that teachers’ own life satisfaction can be a significant factor in their students’ mental health and resilience. Lazarus [144] viewed life satisfaction as a positive appraisal tendency that is related to more effective emotional responses and coping behaviors when facing adversities. Therefore, teachers who are highly satisfied with their lives may provide their students with modelling for a positive outlook, effective coping, and emotional regulation following stress exposure, thereby fostering these qualities among their students as well. In addition, teachers’ positive appraisal tendency may contribute to their own ability to cope and preserve effective functioning under conditions of high stress, which in turn could foster a stable, calm, and supportive haven in an unstable environment. This is consistent with previous findings, which have indicated that teachers with higher life satisfaction manage to create a supportive classroom environment that encourages students to help and share with one another [75].

Contrary to the second hypothesis, no moderating effects emerged for teacher classroom context efficacy on the associations between severity of student armed conflict exposure and student psychiatric symptoms. However, the findings revealed that teacher school context efficacy acts as a protective factor for students’ armed conflict exposure by mitigating their general distress and depression at the between-class level of analysis. In classes in which homeroom teachers experienced low levels of school context efficacy, significant associations were found between class average severity of armed conflict exposure and class average of general distress and depression. These associations did not emerge in classes in which teachers experienced high levels of school context efficacy. This finding regarding general distress should be treated with caution, as it was found to be marginally significant when gender was included in the model. It appears that the inclusion of another variable in the model reduces some of the variability explained by the interaction effect and thus attenuates the interaction effect to be only marginally significant.

A possible explanation for the moderating effect of teacher school context efficacy, together with the non-significant effect of classroom context efficacy, may lie in the differing relevance of these beliefs to teachers’ functioning during armed conflict. Classroom context efficacy typically reflects teachers’ confidence in managing classroom tasks under routine conditions, such as delivering lessons, maintaining a positive learning environment, and supporting students’ moral and social development. However, crises disrupt these routines and introduce new demands and role expectations [145], which may render routine-based competencies less applicable.

For example, exposure to violent events can lead students to become more emotionally reactive and behaviorally dysregulated, as armed conflict has been shown to elicit a range of emotional, cognitive, and behavioral symptoms [29]. Therefore, a teacher’s confidence in their day-to-day classroom management may not align with the complex realities of teaching in crisis situations and, consequently, may not sufficiently support their ability to function effectively. This could offer an explanation for the lack of a moderating effect for classroom context efficacy.

In contrast, school context efficacy appears more relevant in emergency situations. It encompasses teachers’ perceived relationships with colleagues and school leadership, and therefore their opportunities for support, guidance, and consultation. These forms of systemic support not only strengthen teachers’ capacity to respond to students’ needs but also may help sustain their own emotional resilience. Additionally, school context efficacy includes a sense of influence over school-level decision-making and confidence in navigating organizational processes [127]. Teachers who feel empowered within the school system may be better positioned to advocate for students’ needs and contribute to adaptive institutional responses, which is crucial in times of disrupted curricula and heightened uncertainty. Such advocacy may help restore predictability, reassurance, and a sense of safety for students.

Taken together, the significant moderating effects of teacher life satisfaction and school context efficacy, contrasted with the non-significant effect of classroom efficacy, suggest the potential importance of deeper psychological and systemic resources in enabling teachers to support student resilience. While confidence in professional competencies remains essential during routine times, it is likely to be insufficient under extreme conditions. Broader resources, such as school context efficacy and life satisfaction, may help teachers regulate their own emotional responses, sustain professional functioning, and, in return, offer students consistent and supportive interactions.

Moreover, the fact that these moderating effects emerged at the between-class level, but not at the within-class level, suggests that such teacher characteristics influence the classroom in a collective rather than individual manner. Teachers who feel supported may foster a stable and coherent emotional climate that benefits all students, thereby promoting resilience at the group level. Accordingly, teacher well-being and organizational resources should be regarded not only as individual assets, but as essential classroom-wide resources for supporting students’ psychological adjustment during crises.

It is also important to note that the moderating effects of teacher life satisfaction and school context efficacy were not significant with regard to students’ post-traumatic stress symptoms. It may be that teacher life satisfaction and school context efficacy enhance teachers’ emotional availability, enabling them to model adaptive coping or create a more beneficial class environment. These factors, in turn, can enhance the general sense of well-being among students and reduce general symptoms of anxiety, depression, and somatization. However, it appears that these processes are not sufficient to suppress more severe and specific maladaptive reactions to the traumatic exposure, such as post-traumatic stress symptoms, namely, avoidance, hyper-arousal, or emotional numbing. These findings show that teachers may help mitigate students’ general psychological distress symptoms but do not reduce specific post-traumatic stress symptoms, suggesting the limitations of school-based emotional support in addressing severe trauma-related psychopathology. While homeroom teachers appear to play an essential role in fostering a supportive classroom climate that promotes students’ resilience, this role has inherent boundaries. Enduring post-traumatic symptoms are often deeply rooted and typically require targeted, evidence-based clinical interventions. Although teachers can serve as important stabilizing figures and early responders, they are not a substitute for professional mental health care [15]. They can be equipped to identify signs of acute distress and refer students appropriately, but severe psychopathology requires intervention by trained mental health professionals.

### 4.3. Limitations of the Study and Future Directions

To the best of our knowledge, this study is the first to examine the moderating function of teachers’ own well-being and personal resources on their ability to be protective agents promoting student resilience in conditions of armed conflict. However, the findings of the study should be considered in light of several potential limitations that should be addressed in future studies.

The cross-sectional design of the study provides a snapshot of the associations between teacher characteristics and students’ resilience that does not allow for determining the causality in the relations. Longitudinal studies that aid in understanding the directionality of effects between teacher characteristics and student resilience could help in designing interventions that target the relevant mechanisms underlying these relations.

The fact that teachers reported their own life satisfaction and self-efficacy, and students reported their psychiatric symptoms measures, reduced the risk of correlational bias due to shared method variance in the data collected [93]. However, self-report measures have been historically perceived as exposed to social desirability bias [146,147]. Future research should include multi-agent reports and behavioral observations that could strengthen the validity of the results of the present study.

## 5. Conclusions

The main findings of the current study show that teacher life satisfaction and school context efficacy are important factors in teacher ability to be a protective agent in promoting resilience among students. These finding holds insights that may have important implications. From a theoretical stance, these findings provide support for a theoretical shift toward viewing resilience processes from a social-ecology perspective, emphasizing that youth outcomes from traumatic exposure do not occur in a vacuum but are the result of various factors operating across multiple levels of the social environment [47].

The novelty of the current study lies in its focus and identification of teachers’ needs if they are to be a protective agent for students. It sheds light on the importance of promoting teachers’ own welfare and sense of involvement and connectedness in school. The suggestion that the homeroom teachers fulfil the role of support providers to students in times of crisis, can be beneficial for student mental health and is without doubt cost-effective. Still, the findings of the current study suggest that sensitivity, guidance, and most importantly, attention to teachers’ needs, are required when assigning more responsibilities to teachers.

When considering the broader applicability of these findings, it is important to account for cultural variability. Cultural values and norms shape individual expression, relationships, and social roles [148], and research increasingly acknowledges cross-cultural differences in perceptions of teacher–student relationships [149,150,151]. For example, Fabris et al. [151] found that while Italian teachers perceived closeness in terms of emotional empathy and warmth, Chinese teachers associated it more with perceptions of pedagogical effectiveness. Accordingly, culturally shaped perceptions of the teacher–student relationship are likely to influence ways in which teachers support and connect with their students. As a result, culturally different perceptions affect the teacher resources that are considered most relevant and the extent to which these resources can moderate the psychological effects of students’ exposure to adversity. Therefore, before generalizing the present findings or applying them in practice, future research should examine how culturally embedded understandings of teacher–student dynamics shape the teacher’s role as a protective figure.

Our findings demonstrate how school-based contextual factors, particularly the homeroom teacher’s own well-being and school context efficacy, can meaningfully contribute to the prevention of psychopathology in students. The findings point to actionable pathways for school-based interventions that promote teacher well-being and school involvement and connectedness. This is relevant not as outcomes in themselves, but as mechanisms for strengthening teachers’ capacity to enhance student well-being and reduce the risk of emerging mental health difficulties.

The need to empower teachers as a means to support students’ resilience calls for systematic strategies within the school environment. While external constraints may limit resources, school psychologists and school administrators can play a central role by fostering internal practices such as emotional check-ins with teaching staff, staff-building activities, and dedicating a significant portion of the tasks of school psychologists to supporting teachers’ needs [152,153,154]. In situations of environmental adversity that threaten the mental health of all members of the school system, such efforts may provide a cost-effective and impactful way to strengthen both teacher functioning and student well-being.

In conclusion, this study highlights the vital importance of supporting homeroom teachers working in adverse socio-ecological conditions. Tasked with managing classrooms and attending to the emotional needs of distressed students, while contending with their own traumatic exposure, teachers face an exceptionally complex role. Without adequate support, their ability to serve as stabilizing, resilient figures who can emotionally support and guide their students may be compromised. Therefore, any effort to strengthen student mental health in such contexts must include systemic support structures that nurture the psychological and professional resources teachers need to fulfill this role.

## Figures and Tables

**Table 1:** Bivariate correlations between the research variables: unstandardized within-class and between-class coefficients.

	1	2	3	4	5	6	7	8	9	10	11
Within-class level											
1. Student PLE	–	–	–	–	–	–	–	–	–	–	–
2. Student general distress	0.33 ***	–	–	–	–	–	–	–	–	–	–
3. Student anxiety	0.35 ***	0.94 ***	–	–	–	–	–	–	–	–	–
4. Student depression	0.24 ***	0.89 ***	0.78 ***	–	–	–	–	–	–	–	–
5. Student somatization	0.31 ***	0.90 ***	0.80 ***	0.68 ***	–	–	–	–	–	–	–
6. Student PTS symptoms	0.49 ***	0.68 ***	0.68 ***	0.61 ***	0.58 ***	–	–	–	–	–	–
7. Student gender	0.17 ***	0.29 ***	0.30 ***	0.21 ***	0.28 ***	0.22 ***	–	–	–	–	–
M	16.57	0.86	0.96	0.88	0.75	14.64					
SD	10.64	0.84	1.00	0.89	0.87	13.03					
Between-class level											
1. Student PLE	–	–	–	–	–	–	–	–	–	–	–
2. Student general distress	0.28 ***	–	–	–	–	–	–	–	–	–	–
3. Student anxiety	0.38 ***	0.93 ***	–	–	–	–	–	–	–	–	–
4. Student depression	0.19 ***	0.90 ***	0.79 ***	–	–	–	–	–	–	–	–
5. Student somatization	0.15 ***	0.87 ***	0.72 ***	0.67 ***	–	–	–	–	–	–	–
6. Student PTS symptoms	0.65 ***	0.67 ***	0.74 ***	0.50 ***	0.58 ***	–	–	–	–	–	–
7. Teacher SWLS	0.02	−0.28 ***	−0.18 ***	−0.36 ***	−0.24 ***	−0.23 ***	–	–	–	–	–
8. Teacher class efficacy	−0.05 *	−0.26 ***	−0.20 ***	−0.28 ***	−0.24 ***	−0.10 ***	0.20 ***	–	–	–	–
9. Teacher school efficacy	−0.03	−0.03	−0.08 ***	0.002	−0.008	−0.08 ***	0.16 ***	0.31 ***	–	–	–
10. Grade level	0.13 ***	0.20 ***	0.20 ***	0.34 ***	−0.011	−0.01	0.06 *	−0.17 ***	−0.11 ***	–	–
11. Teacher gender	−0.09 ***	−0.15 ***	−0.16 ***	−0.23 ***	0.001	−0.07 **	0.01	0.15 ***	0.01	−0.25 ***	–
12. Teacher seniority	−0.08 **	−0.23 ***	−0.28 ***	−0.26 ***	−0.08 **	−0.18 ***	0.13 ***	0.14 ***	0.21 ***	−0.18 ***	0.39 ***
M	16.57	0.86	0.96	0.88	0.75	14.64	26.64	4.63	4.40		
SD	6.84	0.20	0.25	0.22	0.21	3.87	5.27	0.45	0.63		

* *p* < 0.05, ** *p* < 0.01, *** *p* < 0.001; Note: PLE—Political Life Events; PTS—post-traumatic stress; SWLS—Satisfaction with Life Scale. Student gender coded: 1—male, 2—female; grade level coded: 1—8th grade, 2—9th grade, 3—10th grade, 4—11th grade; teacher gender coded: 1—male, 2—female.

**Table 2:** Student PLE as predictor of student psychiatric symptoms: unstandardized within-class and between-class coefficients.

	Student General Distress	Student Anxiety	Student Depression	Student Somatization	Student PTS Symptoms
Within-class effect					
PLE	0.026 ***	0.032 ***	0.020 ***	0.025 ***	0.608 ***
Between-class effect					
PLE	0.009 *	0.014 **	0.007	0.005	0.382 ***

* *p* < 0.05, ** *p* < 0.01, *** *p* < 0.001; Note: PLE—Political Life Event; PTS—post-traumatic stress.

## Data Availability

The raw data supporting the conclusions of this article will be made available by the authors on request.
